# Man with a Forehead Mass: Detection of a Forehead Pseudoaneurysm with Bedside Ultrasonography

**DOI:** 10.1155/2014/647175

**Published:** 2014-07-24

**Authors:** Amin Abdi, Erick Armijo, Dina Seif, Tarina Kang

**Affiliations:** Department of Emergency Medicine, Los Angeles County + University of Southern California Medical Center, 1200 N. State Street, Suite 1011, Los Angeles, CA 90033, USA

## Abstract

A vascular pseudoaneurysm can present similarly to an abscess; yet incision and drainage of a pseudoaneurysm can lead to uncontrolled bleeding and expose the patient to further morbidity. This is a case of a patient with a forehead pseudoaneurysm who presented to our emergency room after blunt head trauma. Here we review different diagnostic modalities as well as some of the treatment options that are described in the literature.

## 1. Case

**Figure 1:**
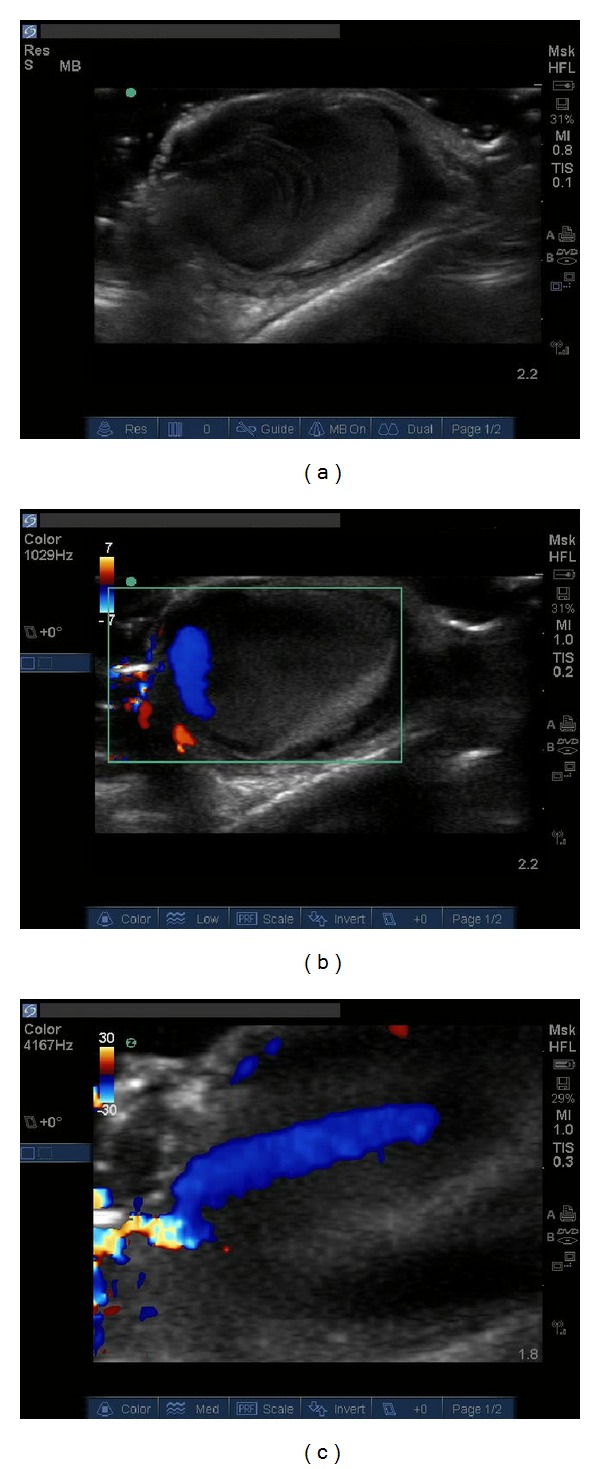
Ultrasound images of patient's pseudoaneurysm. (a) B-mode ultrasonography shows a fluctuant mass with internal echoes similar to an abscess. Dynamic images would reveal wave-like flow through the mass suggestive of communication with a vessel lumen. (b) Color Doppler ultrasonography confirms vascular communication and pulsatile flow. (c) Color Doppler ultrasonography zoomed in on the pseudoaneurysm neck further confirms communication of pseudoaneurysm with an arterial lumen (see Supplementary material which is available online at http://dx.doi.org/10.1155/2014/647175).

A 36-year-old man with a history of alcohol abuse presented to the emergency department (ED) with forehead pain and swelling. The patient had presented 3 weeks earlier with a profusely bleeding forehead laceration after sustaining a ground-level fall. The patient had a normal noncontrast head computed tomography (CT) and the laceration was repaired at that time. Upon subsequent visit, the patient reported worsening forehead pain and swelling. Physical examination revealed a well-circumscribed, fluctuant, erythematous, protuberant mass extending from the forehead. The mass was tender but the overlying laceration was dry and intact with sutures in place. Weak pulsations were palpable. To better elucidate the etiology of the mass a soft tissue bedside ultrasound was performed which showed pulsatile flow within the mass (Figure 1). A CT angiogram of head was performed, which confirmed the presence of a pseudoaneurysm. The head and neck surgery service was consulted for management of the patient's pseudoaneurysm.

## 2. Discussion

A pseudoaneurysm, also known as a false aneurysm, occurs when blood leaks out of a vessel through a defect in the vessel wall. This phenomenon results in a hematoma that remains in communication with the vessel but is contained within the adventitial layer by the surrounding structures [1]. This is unlike a true aneurysm in which all layers of the arterial wall dilate due to congenital (i.e., connective tissue disorders) or acquired defects (i.e., inflammatory vascular diseases such as atherosclerosis). A pseudoaneurysm usually presents as a painful, tender, pulsatile mass. However, it can also be asymptomatic and incidentally diagnosed via routine radiographic or surgical procedures [2]. The rate of pseudoaneurysm expansion depends on the vessel involved and the degree of injury. If left untreated, an enlarged or rapidly expanding pseudoaneurysm can cause ischemia and compression of surrounding structures or rupture and bleed profusely [2]. Pseudoaneurysm formation most commonly occurs as a result of blunt or penetrating trauma to an arterial vessel during central venous catheterization, though any infectious or inflammatory insult to the vessel wall could potentially lead to formation of a pseudoaneurysm [1, 3]. Femoral vein or artery pseudoaneurysms may complicate up to 8% of vascular interventional procedures [3]. There are numerous case reports that describe traumatic pseudoaneurysm formation after blunt or penetrating trauma to the face, trunk, and extremities [4–10].

Traditional angiography performed by an interventional radiologist is still considered the gold standard for diagnosis of pseudoaneurysms [11], though it is being replaced by newer generations of CT angiography (CTA) scanners [12]. CTA has the advantage of being more rapidly and widely available in most emergency departments that do not have interventional radiologists on call [12]. Unlike traditional angiography, CTA is faster and less invasive, can provide detailed information about injury to surrounding structures, and can assist with operative planning [12]. The reported sensitivity and specificity of CTA for detection of pseudoaneurysms are 95.1% and 98.7%, respectively [13]. Yet conventional angiography tends to be used when the CTA is equivocal and the suspicion for pseudoaneurysm remains high [14]. Magnetic resonance angiography (MRA) can also be used to evaluate for pseudoaneurysms. The main advantage of MRA is that it incurs no radiation to the patient [15]. However, MRA is much more expensive and time-consuming and may not be a safe option for patients who have altered mental status or hemodynamic instability [15]. Arterial color and Doppler ultrasonography can assess flow through the pseudoaneurysm and confirm its communication to the vessel lumen. The advantages of ultrasonography include its cost-effectiveness, lack of ionizing radiation, repeatability, and its ability to be performed at the patient's bedside [2]. The role of bedside emergency ultrasound is important in this case because it allowed the clinician to differentiate between an abscess, traumatic hematoma, and pseudoaneurysm. Incision and drainage of a pseudoaneurysm mistaken as an abscess can lead to profuse hemorrhage, which may further complicate the treatment options and necessitate operative intervention and blood product administration [16].

Open surgical ligation is the gold standard treatment for a pseudoaneurysm, though it is the most invasive option [4]. Less invasive management techniques have been described [3, 17–19]. In cases where the pseudoaneurysm is not easily accessible, endovascular stents can be placed over the vessel defect to prevent blood flow through the pseudoaneurysm, causing thrombosis of the blood within the aneurysm. This is a minimally invasive technique that has a high success rate and can be performed by an interventional radiologist or vascular surgeon without the need for an open surgery [3]. Complications of this procedure include stent migration, persistent leakage of blood into the pseudoaneurysm, and infection [17, 18]. Ultrasound-guided thrombin injection is another proposed treatment of pseudoaneurysms, whereby thrombin is directly injected into the pseudoaneurysm allowing it to clot [19]. This technique should not be used if there is an arteriovenous fistula in addition to the pseudoaneurysm, as distal thrombus formation can occur with thrombin injection [19]. Compression of the pseudoaneurysm neck is thought to be the least invasive technique and is recommended for nonobese, nonanticoagulated patients with a narrow pseudoaneurysm neck. Compression with an ultrasound probe for at least 20 minutes prevents blood flow to the aneurysm, allowing blood within the aneurysm to coagulate and regress [3].

In this case, the head and neck surgery service evaluated the patient. The pseudoaneurysm neck was identified using ultrasound and compression therapy was applied. He was observed in the emergency department and had no expansion of his pseudoaneurysm. He was discharged and scheduled for outpatient surgical ligation with no further complications.

## Supplementary Material

Dynamic images of the patient's forehead mass reveals wave-like flow through the mass suggestive of a pseudoaneurysm with persistent communication with a vessel lumen.
